# Manganese Dynamics in Mouse Brain After Systemic MnCl_2_ Administration for Activation-Induced Manganese-Enhanced MRI

**DOI:** 10.3389/fncir.2021.787692

**Published:** 2021-12-20

**Authors:** Hiroki Tanihira, Tomonori Fujiwara, Satomi Kikuta, Noriyasu Homma, Makoto Osanai

**Affiliations:** ^1^Department of Radiological Imaging and Informatics, Tohoku University Graduate School of Medicine, Sendai, Japan; ^2^Faculty of Health and Medical Care, Saitama Medical University, Hidaka, Japan; ^3^Department of Medical Physiology, Faculty of Medicine, Kyorin University, Mitaka, Japan; ^4^Department of Neurophysiology, National Institute of Neuroscience, National Center of Neurology and Psychiatry, Kodaira, Japan; ^5^Department of Intelligent Biomedical Systems Engineering, Graduate Scholl of Biomedical Engineering, Tohoku University, Sendai, Japan; ^6^Laboratory for Physiological Functional Imaging, Department of Medical Physics and Engineering, Division of Health Sciences, Osaka University Graduate School of Medicine, Suita, Japan

**Keywords:** MRI, manganese, whole brain imaging, activity mapping, calcium, non-invasive imaging, neuron

## Abstract

Activation-induced manganese-enhanced MRI (AIM-MRI) is an attractive tool for non-invasively mapping whole brain activities. Manganese ions (Mn^2+^) enter and accumulate in active neurons via calcium channels. Mn^2+^ shortens the longitudinal relaxation time (T1) of H^+^, and the longitudinal relaxation rate R1 (1/T1) is proportional to Mn^2+^ concentration. Thus, AIM-MRI can map neural activities throughout the brain by assessing the R1 map. However, AIM-MRI is still not widely used, partially due to insufficient information regarding Mn^2+^ dynamics in the brain. To resolve this issue, we conducted a longitudinal study looking at manganese dynamics after systemic administration of MnCl_2_ by AIM-MRI with quantitative analysis. In the ventricle, Mn^2+^ increased rapidly within 1 h, remained high for 3 h, and returned to near control levels by 24 h after administration. Microdialysis showed that extracellular Mn returned to control levels by 4 h after administration, indicating a high concentration of extracellular Mn^2+^ lasts at least about 3 h after administration. In the brain parenchyma, Mn^2+^ increased slowly, peaked 24–48 h after administration, and returned to control level by 5 days after a single administration and by 2 weeks after a double administration with a 24-h interval. These time courses suggest that AIM-MRI records neural activity 1–3 h after MnCl_2_ administration, an appropriate timing of the MRI scan is in the range of 24–48 h following systemic administration, and at least an interval of 5 days or a couple of weeks for single or double administrations, respectively, is needed for a repeat AIM-MRI experiment.

## Introduction

The first step in understanding the expression mechanisms of brain functions and pathophysiological mechanisms of neurological disorders is to understand which brain regions are associated with those functions and diseases. To address this issue, we need methods to measure and analyze neural activities within the entire brain volume. Activation-induced manganese-enhanced MRI (AIM-MRI) is one such method for mapping whole brain activities (Lin and Koretsky, 1997; Aoki et al., 2004; Duyn and Koretsky, 2008; Silva and Bock, 2008; Kikuta et al., 2015). Manganese ion (Mn^2+^) can pass through voltage-dependent calcium channels (VDCCs), is taken up by mitochondria and other organelles, binds to proteins, and is extruded very slowly from the cell (Nelson, 1986; Rasagado-Flores et al., 1987; Narita et al., 1990; Low et al., 1991; Nordhøy et al., 2003; Chen et al., 2015). VDCCs open more frequently in highly active neurons; hence, in the presence of Mn^2+^ in the extracellular solution, highly active neurons accumulate larger amounts of Mn^2+^ than weakly active neurons (Kikuta et al., 2015). Therefore, Mn^2+^ is a surrogate marker of Ca^2+^ influx in excitable tissues. A paramagnetic ion, Mn^2+^ shortens the longitudinal relaxation time (T1) of protons (H^+^), and the longitudinal relaxation rate R1 (=1/T1) is proportional to Mn^2+^ concentration ([Mn^2+^]; Nordhøy et al., 2003; Aoki et al., 2004; Silva et al., 2004; Tambalo et al., 2009; Kikuta et al., 2015). Thus, [Mn^2+^] can be quantified by the R1 value calculated from the T1 value (Tambalo et al., 2009). Based on these ideas, AIM-MRI can use R1 to measure neural activity changes in a freely-moving subject with the advantage of being insensitive to blood hemodynamics (Tambalo et al., 2009).

Despite these features, AIM-MRI is still not widely used in the field of neuroscience, partially due to insufficient information regarding Mn^2+^ dynamics in the brain, confounding the interpretation of the results. To resolve this issue, we need to know the dynamics of Mn^2+^ in the brain and to thereby determine three time windows after systemic administration. The first is when Mn^2+^ flows into neurons. Without this timing, it is impossible to record changes in neural activities associated with a specific behavior, task, stimuli, operation, or drug administration. The second is when Mn^2+^ in the extracellular space has cleared but is still maintained intracellularly. As MRI cannot discriminate between intracellular and extracellular spaces, the R1 map should be acquired during this time to record neural activities. The third time window is the interval required for repeated measurements. Because MRI is a non-invasive method, one of its merits is repeated measurements within the same subject. To conduct AIM-MRI repeatedly in a single subject, [Mn^2+^] in the brain must have returned to its control level when the next dose of MnCl_2_ is administered. Another reason to know when [Mn^2+^] in the brain returns to its normal concentration for repeated measurements is that, although Mn^2+^ is an essential heavy metal (Horning et al., 2015), overexposure has some toxic consequences in neuronal and non-neuronal tissues (Hazell, 2002; Silva and Bock, 2008; Chen et al., 2015; Bouabid et al., 2016). Mn^2+^ binds to many proteins and accumulates in organelles; thus, the clearance of manganese from brain tissue is very slow (Takeda et al., 1995). To determine these three time windows, we conducted a longitudinal study and estimated the time courses of [Mn^2+^] in extra- and intracellular-spaces after systemic administration of MnCl_2_ by AIM-MRI with quantitative T1 measurement and microdialysis.

## Materials and Methods

### Animals

We used 8 to 16-week-old male C57BL/6 mice (Clea Japan). All mice were maintained at 22–24^°^C on a 12-h light/dark cycle and permitted *ad libitum* access to food and water. The Tohoku University Committee for Animal Experiments and the Kyorin University Animal Care Committee approved all animal experiments, and the experiments were performed in accordance with the Guidelines for Animal Experiments and Related Activities of Tohoku University and Kyorin University, as well as the guiding principles of the Physiological Society of Japan and the National Institutes of Health, United States.

### MRI Acquisition and Image Analysis

Acquisition and analysis methods for MRI data were similar to those previously described (Kikuta et al., 2015; Inami et al., 2019). Mice were injected with MnCl_2_ solutions (0.2 mmol kg^–1^ in saline, i.p.) once or twice at 24-h intervals. MRIs were acquired before and after MnCl_2_ administrations using an AV 400 WB 9.4-T, 89-mm spectrometer equipped with a 150 G/cm gradient insert (Bruker), and an 18-mm 1H volume coil (Takashima Seisakusho) was used for transmission and signal detection. After pre-anesthesia with 3–4% isoflurane (Pfizer), mice were positioned in the MRI scanner and maintained at 1–2% isoflurane in a mixture of air and O_2_ (air:O_2_ = 8:2) using a nose cone. Body temperature was maintained by the circulation of heated water. For T1 measurements in the brain, rapid acquisition with relaxation enhancement (RARE) with a variable repetition time (RARE-VTR) pulse sequence with 7 repetition times (TR: 540, 900, 1,200, 2,000, 3,000, 4,500, and 7,500 ms) was used with an effective echo time (TE_eff_) = 7 ms, RARE factor = 2, matrix size = 160 × 140, field-of-view (FOV) = 16 × 14 mm^2^, slice thickness = 0.5 mm, number of slices = 20, and NEX (number of averages) = 2. Multislice, fast spin-echo T_2_-weighted images (RARE, TE_eff_ = 30 ms, TR = 3,000 ms, RARE factor = 4, NEX = 3) were acquired and used to co-register images to the mouse brain T2-weighted template image that was acquired in advance (Kikuta et al., 2015). Total time in the MRI scanner was about 1 h, and mice were then returned to their home cage.

After spatial filtering, parametric T1 maps were calculated pixel-by-pixel by fitting with the following equation using ParaVision 5.1 software (Bruker):


SI(TR)=A-Bexp(-TR/T1),

where SI(TR) is the signal intensity of each pixel at a given TR.

Pixels in which T1 values were longer than 6,000 ms or shorter than 500 ms were excluded from the analysis. The T2-weighted images were registered to the T2-weighted template image, and the T1 maps were co-registered simultaneously using SPM12 software (Wellcome Trust Centre for Neuroimaging, University College London). Because R1 is proportional to [Mn^2+^], R1 maps were calculated pixel-by-pixel by inverting T1 values of the T1 map to visualize [Mn^2+^] distribution. [Note: As R1 is less sensitive to Mn^3+^, we treated R1 as reflecting Mn^2+^ (Topping et al., 2017)].

The R1 value of the ROI was calculated by averaging the T1 of each pixel in the ROI on the T1 map, and then inverting the T1 value of the ROI. To determine the ROIs, we used the mouse brain atlas (2014 Allen Institute for Brain Science, Allen Mouse Brain Atlas, http://mouse.brain-map.org/) registered to the template image (Kikuta et al., 2015) and queried the structures from the brain atlas.

Non-uniform R1 distribution may be caused by a non-uniform magnetic field such as the edge of the coil. To avoid this, we always kept the distance between the center of the imaging volume and the isocenter of the MRI scanner less than 1 mm. We also checked the heterogeneity in R1 distribution due to problems of the MRI scanner using an agarose gel phantom. The distribution of R1s in the gel phantom was uniform (data not shown).

### *In vivo* Microdialysis

Male mice (12–15 weeks old; a total of 6 mice) were anesthetized with a combination anesthetic comprised of medetomidine (0.3 mg/kg; Orion Pharma), midazolam (4.0 mg/kg; Astellas Pharma), and butorphanol (5.0 mg/kg; Meiji Seika Pharma). The mice were placed in a stereotaxic apparatus (Narishige), and a guide cannula was unilaterally implanted in the striatum (bregma coordinates: anteroposterior, 0.8 mm; mediolateral, 1.8 mm; and dorsoventral, 2.5 mm) according to the mouse brain atlas (Paxinos and Franklin, 1997) and fixed to the skull with dental cement. Antisedan (1.0 mg/kg; Orion Pharma) was then administrated to recover from the anesthesia, and the mice were placed individually in their home cage. 3 days after the surgery, a dialysis probe (membrane length 1 mm; Eicom) was inserted into the guide cannula, and saline containing 0.005% BSA was perfused at a constant flow rate (1 μL/min) using a micro-syringe pump (Eicom). The dialysates were collected at 60 min/fraction. To quantify the [Mn^2+^] in the extracellular solution, the dialysates were analyzed with an inductively coupled plasma-mass spectrometer (ICP-MS, Agilent 8800, Agilent technologies). After the microdialysis study, mice were sacrificed under deep anesthesia with pentobarbital (60 mg/kg), the brains were obtained and fixed with paraformaldehyde. Serial coronal sections were prepared and used to determine the placement site of the dialysis probe. As the pre-MnCl_2_ administration [Mn] in the dialysates was different in each animal, changes in [Mn] after MnCl_2_ administration were represented as the difference between pre- and post- MnCl_2_ administration [Mn] and expressed as Δ[Mn].

### Statistical Analysis

Statistical analyses were performed using JMP Pro 15 (SAS Institute), MATLAB (Mathworks), and SPM12 software (Wellcome Trust Centre for Neuroimaging, University College London). In the statistical parametric mapping (SPM) analysis, the threshold of significance at the voxel level was set at *P* < 0.025 because the SPM12 software performs a one-tailed Student’s *t*-test. For other statistical tests, differences with a *P* value of less than 0.05 were considered significant. All data are presented as mean ± s.e.m unless stated otherwise.

## Results

### Mn^2+^ Dynamics After Single MnCl_2_ Administration

**FIGURE 1:**
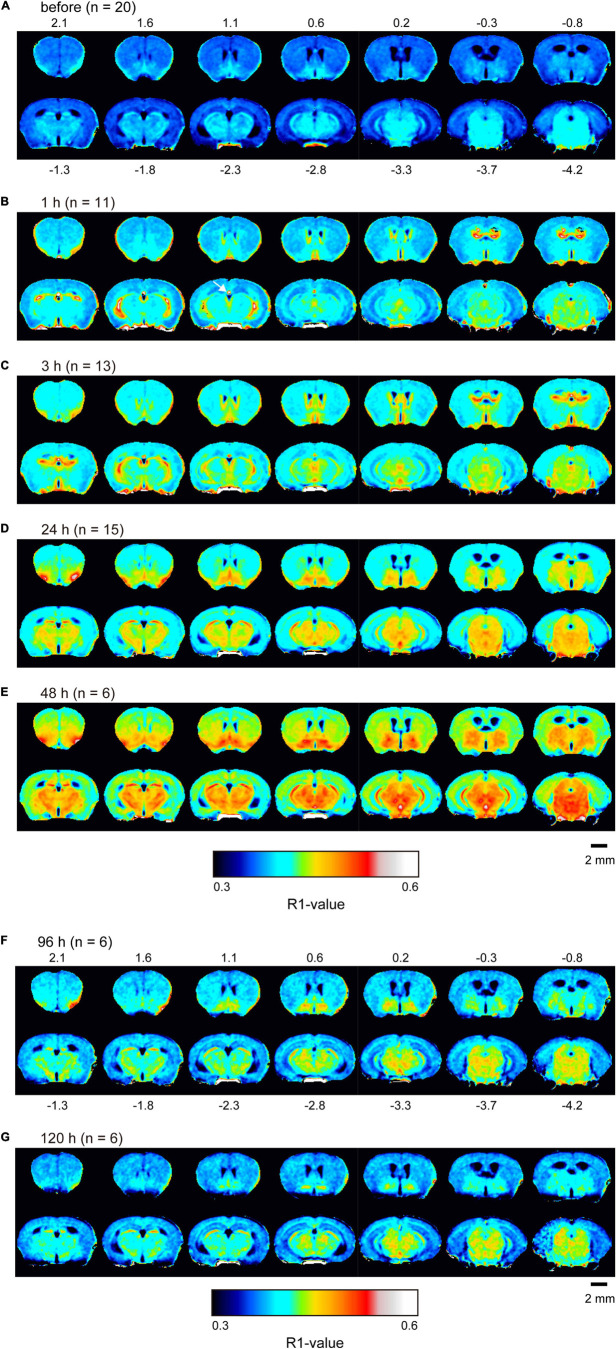
Mn^2+^ distribution in the brain before and after single MnCl_2_ administration represented by R1 maps. The pseudo-color maps represent the distribution of mean R1 values before **(A)**, 1 h **(B)**, 3 h **(C)**, 24 h **(D)**, 48 h **(E)**, 96 h **(F)**, and 120 h **(G)** after administration. Pixels whose R1 value did not exceed three times the standard deviation are shown in black. The number of mice at each time is indicated as *n*. Numbers above and below each slice in **(A,F)**, respectively, indicate the anterior-posterior coordinates from bregma. The arrow in panel **(B)** indicates the superior part of the third ventricle, which exhibited a high R1 value (Supplementary Figure 2).

**FIGURE 2:**
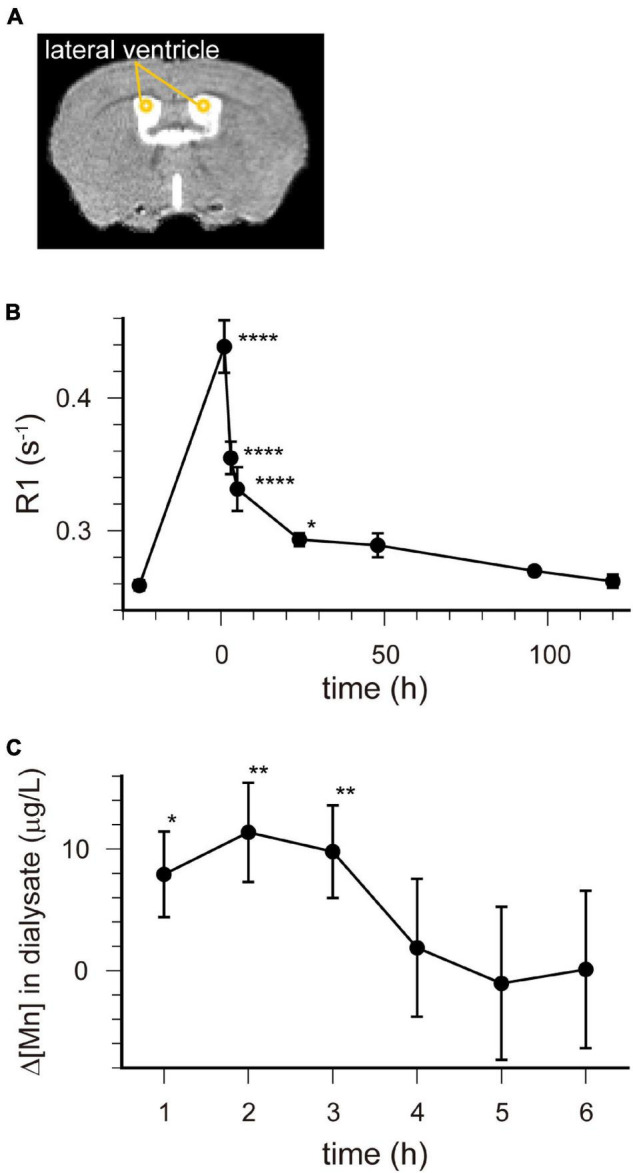
[Mn^2+^] dynamics in the extracellular fluid following a single administration of MnCl_2_. **(A)** The locations of ROIs are indicated on the template MRI image. **(B)** Time course of the R1 values in the lateral ventricle. The horizontal axis indicates the time following MnCl_2_ administration. Each point represents the R1 values before (*n* = 20), and 1 (*n* = 11), 3 (*n* = 13), 5 (*n* = 7), 24 (*n* = 15), 48 (*n* = 6), 96 (*n* = 6), and 120 (*n* = 6) hours after the administration. *P < 0.05, ****P < 0.0001 (Dunnet test, compared with R1 values before MnCl_2_ administration). These values are also shown in Table 1. **(C)** Time course of the change in Mn concentration (Δ[Mn]) in the extracellular space of the striatum compared with pre-administration. Each data point represents the data smoothed by the moving bin method with bin size 2. *P < 0.05, **P < 0.025 (*n* = 6, one-tailed one-sample *t*-test).

R1 maps reflecting [Mn^2+^] (Figure 1) showed that in the lateral and third ventricles, R1 values increased quickly and peaked 1 h after MnCl_2_ administration (Figures 1B,2A,B and Supplementary Figures 1A, 2). R1 then decreased within 5 h following administration but remained higher than the control value for at least 24 h after the administration (Figure 2B, Supplementary Figure 2, and Table 1). Although the absolute R1 values of the superior and inferior third ventricles were different (Figure 1B arrow, Supplementary Figure 2), both time courses were similar to that of the lateral ventricle. The differences in the absolute R1 values among the various cerebral ventricles may be due to differences in their contents of choroid plexus (see section “Discussion”). The ventricular R1 reflects the [Mn^2+^] of cerebrospinal fluid (CSF), but it is not known whether this reflects [Mn^2+^] in the extracellular fluid. To confirm this, we quantified the [Mn^2+^] in extracellular fluids via microdialysis (Figure 2C and Supplementary Figure 3). In the dialysates, [Mn] increased rapidly within 1 h and remained high until 3 h after administration, whereupon it returned to control levels 4 h after administration. Mn^2+^ is known to be excreted in bile (Hurley and Keen, 1987). To confirm whether the decrease in [Mn^2+^] in the CSF was due to its excretion from the body, we evaluated the signal intensity of the T1-weighted images of the gallbladder (Supplementary Figure 4). The time course of signal intensity of the T1-weighted images resembled those of the R1s in the ventricles. Significant differences in the normalized signal intensities were observed at 1, 3, and 5 h after MnCl_2_ administration compared with those before the administration. To summarize, these results indicate that extracellular [Mn^2+^] increased immediately after systemic administration of MnCl_2_, remained high for 1–3 h, and returned to control levels 24–48 h after administration via excretion.

**TABLE 1:** R1 values [×10^–1^ (s^–1^)] in brain regions following single MnCl_2_ administration.

Time (h)	Region								
	Lateral ventricle	SM-ctx	Vis-ctx	Str	Thal	SI	SC	IPN	*n*
Pre	2.59 ± 0.04	3.58 ± 0.05	3.54 ± 0.05	3.62 ± 0.06	3.76 ± 0.08	3.83 ± 0.07	3.90 ± 0.07	4.01 ± 0.08	20
1	4.39 ± 0.20*^d^*	3.77 ± 0.07	3.78 ± 0.08*^a^*	3.86 ± 0.09	3.99 ± 0.12	4.05 ± 0.11	4.15 ± 0.10	4.28 ± 0.14	11
3	3.55 ± 0.12*^d^*	3.93 ± 0.06*^d^*	3.92 ± 0.07*^b^*	4.00 ± 0.07*^c^*	4.15 ± 0.09*^b^*	4.17 ± 0.09*^a^*	4.22 ± 0.08*^a^*	4.54 ± 0.15*^b^*	13
5	3.31 ± 0.17*^d^*	3.82 ± 0.11	3.89 ± 0.08*^a^*	3.96 ± 0.12*^a^*	4.10 ± 0.15	4.15 ± 0.15	4.22 ± 0.13	4.49 ± 0.21	7
24	2.93 ± 0.05*^a^*	4.04 ± 0.04*^d^*	4.06 ± 0.04*^d^*	4.18 ± 0.06*^d^*	4.40 ± 0.07*^d^*	4.66 ± 0.06*^d^*	4.52 ± 0.06*^d^*	4.79 ± 0.10*^d^*	15
48	2.89 ± 0.09	4.21 ± 0.10*^d^*	4.24 ± 0.09*^d^*	4.41 ± 0.11*^d^*	4.71 ± 0.13*^d^*	5.05 ± 0.14*^d^*	4.82 ± 0.14*^d^*	5.17 ± 0.14*^d^*	6
96	2.70 ± 0.03	3.79 ± 0.04	3.76 ± 0.03*^a^*	3.90 ± 0.03	4.13 ± 0.03	4.48 ± 0.04*^c^*	4.37 ± 0.02*^b^*	4.56 ± 0.06*^a^*	6
120	2.62 ± 0.05	3.72 ± 0.03	3.76 ± 0.03	3.77 ± 0.03	4.06 ± 0.03	4.12 ± 0.11	4.36 ± 0.04*^b^*	4.44 ± 0.03	6

*Values are presented as means ± SEM. Numbers of animals are indicated as n. Superscripts indicate statistically significant levels compared with pre-administration R1 values (^a^P < 0.05; ^b^P < 0.01; ^c^P < 0.001; and ^d^P < 0.0001, Dunnett test).*

*SM-ctx, sensorimotor cortex; Vis-ctx, visual cortex; Str, striatum; Thal, thalamus; SI, substantia innominata; SC, superior colliculus; and IPN, interpeduncular nucleus.*

**FIGURE 3:**
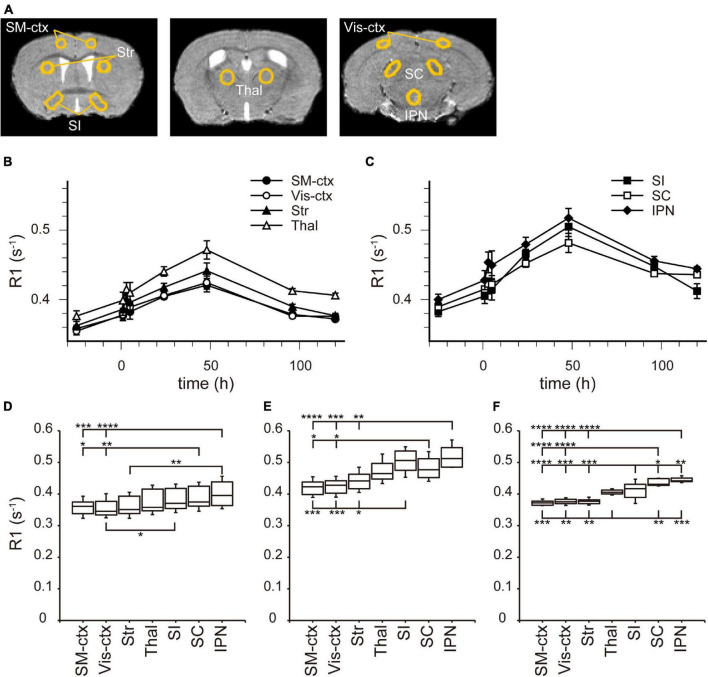
[Mn^2+^] dynamics in the brain parenchyma after a single MnCl_2_ administration. **(A)** The locations of ROIs are indicated on the template MRI image. **(B,C)** Time courses of R1 values from the ROIs in **(A)**. **(B)** R1 values in SM-ctx, Vis-ctx, Str, and Thal, and **(C)** those in SI, SC, and IPN. The R1 values and statistical differences from control are shown in Table 1. Each point represents the R1 values before (*n* = 20), and 1 (*n* = 11), 3 (*n* = 13), 5 (*n* = 7), 24 (*n* = 15), 48 (*n* = 6), 96 (*n* = 6), and 120 (*n* = 6) hours after the administration. **(D–F)** The R1 values before **(D)**, 48 h **(E)**, and 120 h **(F)** after single MnCl_2_ administration in each brain region. *P < 0.05, **P < 0.01, ***P < 0.001, and ****P < 0.0001 (Tukey–Kramer test). SM-ctx, sensorimotor cortex; Vis-ctx, visual cortex; Str, striatum; Thal, thalamus; SI, substantia innominata; SC, superior colliculus; and IPN, interpeduncular nucleus.

In the brain parenchyma, the regions showing higher R1 values gradually spread from the ventricles to almost all brain regions 24 h after MnCl_2_ administration (Figures 1C,D and Supplementary Figures 1B,C). The time courses of R1 in sensorimotor cortex, visual cortex, striatum (Str), thalamus (Thal), substantia innominata (SI), superior colliculus (SC), and interpeduncular nucleus (IPN), are shown in Figure 3 and Table 1. The R1 values slowly increased compared with those in the ventricles, peaked at 48 h after MnCl_2_ administration, and then gradually decreased, returning to control levels 120 h after the administration, except in the caudal subcortical regions, such as SC (Figures 1, 3B,C, Supplementary Figure 1, and Table 1). The distribution of R1 was non-uniform. Before and 48 h after MnCl_2_ administration, subcortical regions, such as IPN, SC, and SI exhibited higher R1 values than those in the cortex and the Str (Figures 3D,E). At 120 h after administration, R1 values in the subcortical regions remained higher than those in the cortex and the Str (Figure 3F).

### Mn^2+^ Dynamics After Double MnCl_2_ Administration

In some AIM-MRI studies, MnCl_2_ was administered several times to maintain a high enough [Mn^2+^] to be detected by MRI, but at the same time considering its toxicity (Bock et al., 2008; Tambalo et al., 2009; Kikuta et al., 2015). To understand the behavior of Mn^2+^ in the brain following repeated MnCl_2_ administration, we also analyzed R1 in the brain after double MnCl_2_ injections (0.2 mmol kg^–1^, i.p. at 24-h intervals).

**FIGURE 4:**
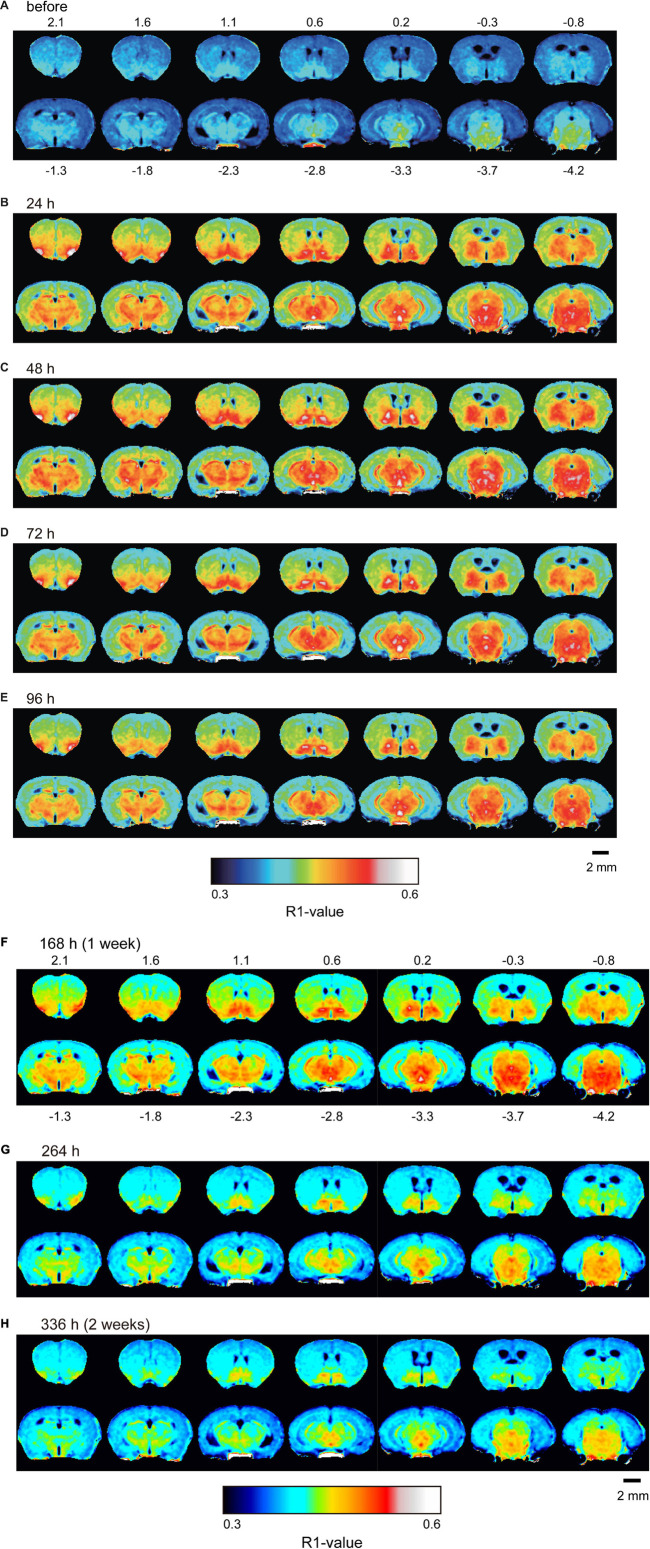
Mn^2+^ distribution in the brain before and after double MnCl_2_ administration represented by R1 maps. The pseudo-color maps represent the distribution of mean R1 values before **(A)**, 24 h **(B)**, 48 h **(C)**, 72 h **(D)**, 96 h **(E)**, 168 h **(F)**, 264 h **(G)**, and 336 h **(H)** after the administrations. The illustrations are similar to those in Figure 1. *n* = 4 for all conditions.

**FIGURE 5:**
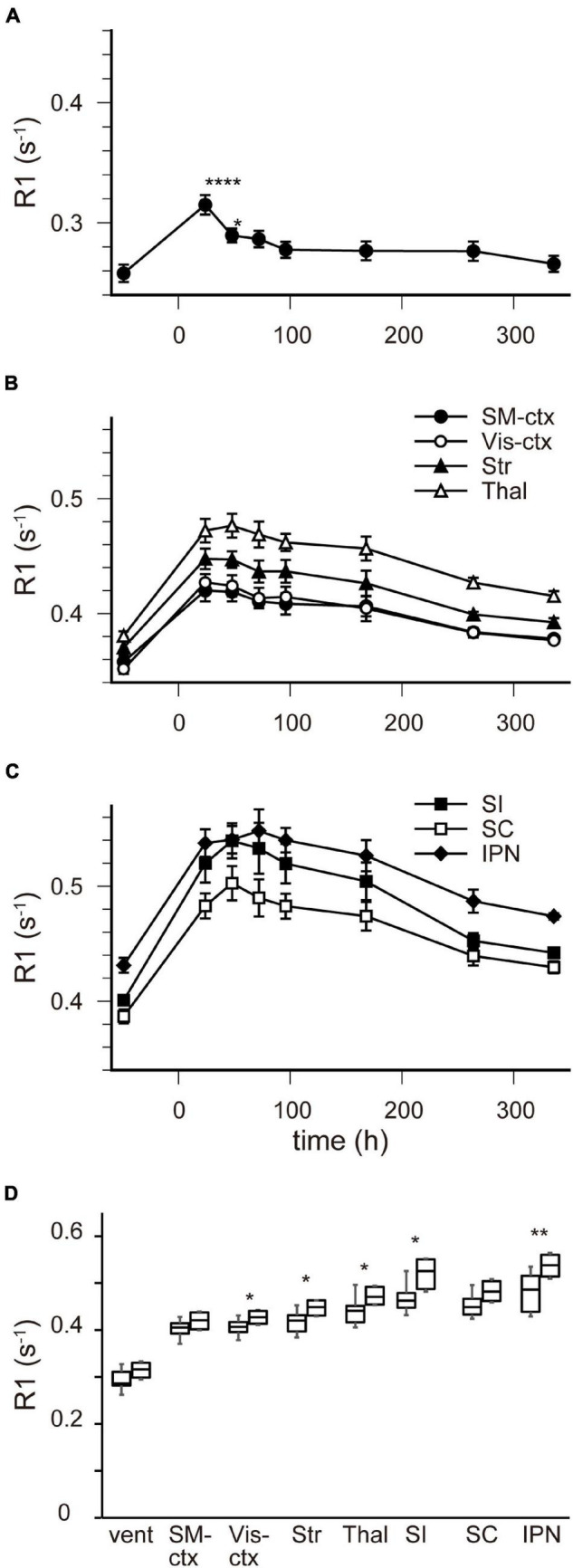
[Mn^2+^] dynamics in the brain following a double MnCl_2_ administration. **(A–C)** Time courses of R1 values in the ventricles **(A)**, in SM-ctx, Vis-ctx, Str, and Thal **(B)**, and in SI, SC, and IPN **(C)**. The R1 values and statistical differences from control are shown in Table 2. Each point represents the R1 values before, and 24, 48, 72, 96, 168, 264, and 336 h after the administration. **(D)** Comparisons of R1 values in each brain region 24 h following single and double administrations. The left-hand bars in each region indicate the R1 value following single administrations and the right-hand ones are following double administration. *P < 0.05, **P < 0.01, ****P < 0.0001 (unpaired two-tailed *t*-test). **(A–C)** *n* = 4 for all conditions. **(D)** *n* is shown in Table 1 and 2. vent, lateral ventricle; SM-ctx, sensorimotor cortex; Vis-ctx, visual cortex; Str, striatum; Thal, thalamus; SI, substantia innominata; SC, superior colliculus; IPN, interpeduncular nucleus.

Figure 4 shows the time course of the distribution of R1. These R1 maps were quantitatively assessed by SPM (Supplementary Figure 5), and the time courses of R1 in several regions are shown in Figure 5 and Table 2. Ventricular R1 returned to nearly control levels 48 h after administration, and by 72 h there was no difference from pre-administration levels (Figure 5A and Table 2). The R1 values in the brain parenchyma peaked 24–48 h after the second administration, resembling the dynamics following a single administration. However, these values remained high for a longer period than those following a single administration, and it took at least 2 weeks (336 h) until they returned to control levels (Figures 4, 5B,C, Supplementary Figure 5, and Table 2). Similar to the single administration, the distribution of R1 was non-uniform, with the subcortical regions exhibiting higher R1 values (Figure 4 and Supplementary Figure 5). Comparisons of the R1 values 24 h after the single and double administrations showed significant differences in several brain parenchymal regions, especially in the subcortical regions (Figure 5D).

**TABLE 2:** R1 values [×10^–1^ (s^–1^)] in brain regions following double MnCl_2_ administration (*n* = 4).

Time (h)	Region							
	Lateral ventricle	SM-ctx	Vis-ctx	Str	Thal	SI	SC	IPN
Pre	2.58 ± 0.07	3.58 ± 0.06	3.52 ± 0.04	3.70 ± 0.06	3.81 ± 0.04	4.01 ± 0.04	3.87 ± 0.06	4.31 ± 0.07
24	3.15 ± 0.08*^d^*	4.20 ± 0.10*^d^*	4.27 ± 0.07*^d^*	4.48 ± 0.09*^d^*	4.72 ± 0.10*^d^*	5.20 ± 0.17*^d^*	4.83 ± 0.11*^d^*	5.38 ± 0.12*^d^*
48	2.89 ± 0.06*^a^*	4.19 ± 0.08*^d^*	4.24 ± 0.09*^d^*	4.47 ± 0.07*^d^*	4.77 ± 0.10*^d^*	5.39 ± 0.15*^d^*	5.03 ± 0.15*^d^*	5.40 ± 0.12*^d^*
72	2.86 ± 0.07	4.11 ± 0.05*^c^*	4.13 ± 0.09*^d^*	4.37 ± 0.10*^d^*	4.69 ± 0.11*^d^*	5.33 ± 0.22*^d^*	4.90 ± 0.16*^d^*	5.48 ± 0.19*^d^*
96	2.78 ± 0.07	4.08 ± 0.09*^c^*	4.14 ± 0.09*^d^*	4.37 ± 0.10*^d^*	4.62 ± 0.07*^d^*	5.20 ± 0.17*^d^*	4.83 ± 0.11*^d^*	5.40 ± 0.11*^d^*
168	2.77 ± 0.08	4.06 ± 0.09*^c^*	4.04 ± 0.11*^c^*	4.26 ± 0.11*^c^*	4.57 ± 0.10*^d^*	5.04 ± 0.16*^c^*	4.74 ± 0.13*^d^*	5.27 ± 0.13*^d^*
264	2.76 ± 0.08	3.84 ± 0.04	3.83 ± 0.04*^a^*	3.99 ± 0.02	4.27 ± 0.04*^b^*	4.52 ± 0.07	4.39 ± 0.08*^a^*	4.87 ± 0.10*^a^*
336	2.66 ± 0.07	3.78 ± 0.03	3.77 ± 0.02	3.92 ± 0.04	4.15 ± 0.04*^a^*	4.42 ± 0.04	4.29 ± 0.05	4.74 ± 0.03

*Values are presented as means ± SEM. Super scripts indicate statistically significant levels compared with pre-administration R1 values (^a^P < 0.05; ^b^P < 0.01; ^c^P < 0.001; and ^d^P < 0.0001, Dunnett test).*

*SM-ctx, sensorymotor cortex; Vis-ctx, visual cortex; Str, striatum; Thal, thalamus; SI, substantia innominata; SC, superior colliculus; and IPN, interpeduncular nucleus.*

## Discussion

Understanding the behavior of Mn^2+^ in the brain after systemic administration of MnCl_2_ for AIM-MRI studies is crucial for optimizing experimental protocols and improving the reliability of the data obtained. In this study, we aimed to determine (1) when Mn^2+^ flows into neurons, (2) when Mn^2+^ in the extracellular space has been cleared while maintaining intracellular Mn^2+^, and (3) what is the appropriate interval required for repeated measurements. Although other studies have evaluated the distribution of Mn^2+^ in the brain after systemic administration of MnCl_2_ (Takeda et al., 1995; Kuo et al., 2005; Tambalo et al., 2009; Chan et al., 2017; Topping et al., 2017), the above three time windows have not been previously clarified.

Ventricular R1 rapidly increased and peaked 1 h after administration, and then rapidly decreased. However, while the difference between the pre-administration R1 values and those at 5 h were 40% of those at 1 h, they were still significantly different from control and they remained a little bit higher than controls for at least 24 h (Figures 1, 2B and Table 1). The ventricular R1 time course reported by Kuo et al. (2005) was similar to ours, although they only showed data for 15 min, 45 min, 24 h, and 72 h after administration. The time course of [Mn] in the extracellular fluid obtained by microdialysis was similar to that of the ventricular R1 (Figure 2C), which is consistent with previous studies (Tseng et al., 2003; Chung et al., 2007). Therefore, the time course of the ventricular R1 should reflect the [Mn^2+^] in the extracellular fluid. However, microdialysis showed that the [Mn^2+^] returned to control levels 4 h after administration, but ventricular R1 was significantly different from the control levels until 24 h. This difference may be attributed to the fact that it is difficult to detect Mn bound to macromolecules such as proteins by microdialysis, while MRI can detect this. To summarize, these results suggest that the time window for Mn^2+^ influx into neurons is in the range of 1–3 h after MnCl_2_ administration.

The absolute R1 values of the lateral ventricle and the superior and inferior third ventricles differed from one another (Figure 2B and Supplementary Figure 2). The choroid plexus seems to be more abundant in the superior dorsal third ventricle and less abundant in the inferior one (Paxinos and Franklin, 1997). It is known that the absolute value of R1 is lower in free water such as CSF than in bound water such as intracellular fluids and free water is less sensitive to Mn^2+^ than intracellular fluids (Nordhøy et al., 2003). The region of the superior third ventricle contains the intracellular fluid of the choroid plexus, which may have resulted in the higher value of R1. The R1 values of the lateral ventricle were between those of the superior and inferior parts of the third ventricle. This may indicate that the ROI set for the lateral ventricle includes the choroid plexus to some extent. Altogether, although the possibility remains that [Mn^2+^] may vary from place to place, the fact remains that after intraperitoneal administration of MnCl_2_, Mn^2+^ in the CSF rises first, followed by that in the brain parenchyma.

Circulating Mn^2+^ dynamics are an important factor for explaining Mn^2+^ dynamics. According to the literature, the time course of R1 in the blood after intraperitoneal administration is similar to the ventricular R1 we observed (Jasmin et al., 2021) and systemic administration of MnCl_2_ by intravenous and intraperitoneal routes induced similar time courses of T1 (inverse of R1) changes in the mouse brain (Kuo et al., 2005). The time course of the signal intensity in the gallbladder obtained by T1-weighted images also resembled that of the ventricular R1 (Supplementary Figure 4). Therefore, MnCl_2_ administered by intraperitoneal injection is likely transported to the CSF through the circulatory system and excreted from the body via the circulatory system to the bile.

In the brain parenchyma, the R1 values increased more slowly than in the ventricle, reaching peak values 24–48 h after both single and double administrations of MnCl_2_ (Figures 1, 3–5, Supplementary Figures 1, 5, and Tables 1, 2). As the R1 values in the ventricles, which reflect extracellular [Mn^2+^], had returned to control levels 24–48 h after MnCl_2_ administration, those in the brain parenchyma during this time should reflect intracellular [Mn^2+^]. Taken together, these findings indicate that 24–48 h after MnCl_2_ administration is an appropriate time window for MRI examination.

Despite the decrease in [Mn^2+^] in the CSF, manganese concentration in the brain parenchyma continued to increase until 24–48 h after MnCl_2_ administration. There are three possible reasons for this. The first is that the R1 of CSF is less sensitive to Mn^2+^ and thus underestimates [Mn^2+^] in the ventricles, the second is that the cells have an active mechanism to take up manganese such as the transferrin transport system (Chen et al., 2015; Horning et al., 2015), and the third is that intracellular manganese binds easily to proteins and thus a small amount of extracellular Mn^2+^ continues to enter to the cells.

We also followed the R1 values until they returned to control levels. After the single and double administration of MnCl_2_, it took 5 days and 2 weeks, respectively, for parenchymal R1 values to return to near control values (Figures 3, 5, Supplementary Figures 1, 5, and Tables 1, 2). However, some regions, such as the SC and Thal exhibited slightly higher R1 values compared to the control R1. The residual [Mn^2+^] calculated from the residual R1 values using the relaxivity 5.35 mM^–1^ s^–1^ obtained from the same MRI scanner (Kikuta et al., 2015) is on the order of several μM. It is reported that the tissue contents of Mn in mammals are several to several tens μM. Therefore, the toxicity of the residual Mn^2+^ is quite small. Nevertheless, because the accumulation of Mn^2+^ through repeated experiments might be toxic, it is desirable to have a longer interval. Therefore, the interval between AIM-MRI experiments should be more than at least 5 days or 2 weeks for single or double administrations, respectively, for repeated AIM-MRI experiments in the same animal, thus avoiding both the effects of residual Mn^2+^ and its toxicity.

The distribution of parenchymal R1 was non-uniform (Figures 1, 4). It is not possible to determine by MRI whether these differences are due to differences in Mn^2+^ accumulation or differences in biochemical environments that may affect the relaxivity of Mn^2+^ (Topping et al., 2017). Therefore, we cannot determine the distribution of neural activity in the brain by the R1 distribution pattern in a single subject. To identify areas where changes in neural activity are seen under certain conditions, comparisons with control animals given the same amount of Mn^2+^ are necessary. It is noted that the non-uniformity of R1 might arise from the scanning quality of the MRI scanner. Thus, it is necessary that the scanning quality should be ensured (see section “Materials and Methods”).

In the case of the double administration, R1 values 24 h after administration were significantly higher in many brain regions than they were following a single administration (Figure 5D). Whereas the cumulative dosage of the double administration is twice as much as that of the single administration, the differences in R1 values between the single and the double administration were small. When considering Mn^2+^ toxicity, a single dose is more suitable for measuring changes in brain activity associated with brief stimuli or tasks using AIM-MRI, while repeated administration is suitable for capturing changes in persistent brain activity, such as in neurological disorders (Kikuta et al., 2015), chronic pain (Inami et al., 2019), and visualization of active polysynaptic circuits (Almeida-Corrêa et al., 2018).

In many AIM-MRI studies, activity-dependent Mn^2+^ accumulation in neurons is assessed by T1-weighted images and quantified by the signal intensities (Lin and Koretsky, 1997; Aoki et al., 2004; Schroeder et al., 2016; Devonshire et al., 2017). However, the signal enhancement can be unreliable in inter-animal comparisons (Tambalo et al., 2009). In addition, the signal intensity does not always correspond to [Mn^2+^], because Mn^2+^ shortens the transverse relaxation time (T2), leading to a decrease in signal intensity associated with [Mn^2+^] (Cory et al., 1987; Pels Rijcken et al., 1994; Silva et al., 2004). Therefore, we measured T1 values instead of the signal intensity of T1-weighted images in the brain and calculated R1 values to quantify the [Mn^2+^].

As mentioned in the introduction, Mn^2+^ is known to be a surrogate marker for Ca^2+^, hence AIM-MRI can measure changes in neural activity by evaluating the R1 map. However, we need to be aware that Mn^2+^ can pass through not only VDCCs but also other Ca^2+^-permeable channels, such as some ligand-gated channels and store-operated calcium channels (Uehara et al., 2002; Itoh et al., 2008; Tu et al., 2009; Chen et al., 2015; Bouabid et al., 2016; Kikuta et al., 2019). The inositol 1,4,5-trisphosphate receptor on the endoplasmic reticulum membrane is also permeable to Mn^2+^ (Striggow and Ehrlich, 1996). Therefore, we should take into consideration that the cellular accumulation of Mn^2+^ may not only depend on neural activity but also on changes in extracellular ligand concentration and intracellular signal transduction. It is known that glial cells also accumulate Mn^2+^ (Tholey et al., 1990; Kikuta et al., 2015). Taken together, these findings suggest that R1 may be affected not only by neural activities but also by other physiological signals such as metabolic changes.

The three time windows of Mn^2+^ dynamics that we have determined should enable neuroscientists to better optimize their experimental protocols for AIM-MRI and to improve the reliability of the data they obtain. Thus, our results pave the way for AIM-MRI to be more widely used in the field of neuroscience.

## Data Availability Statement

The original contributions presented in the study are included in the article/Supplementary Material, further inquiries can be directed to the corresponding author/s.

## Ethics Statement

The animal study was reviewed and approved by The Tohoku University Committee for Animal Experiments and The Kyorin University Animal Care Committee.

## Author Contributions

HT, TF, SK, and MO conceived and performed experiments. HT, TF, and MO analyzed data. HT, TF, NH, and MO wrote the manuscript. All authors contributed to the article and approved the submitted version.

## Conflict of Interest

The authors declare that the research was conducted in the absence of any commercial or financial relationships that could be construed as a potential conflict of interest.

## Publisher’s Note

All claims expressed in this article are solely those of the authors and do not necessarily represent those of their affiliated organizations, or those of the publisher, the editors and the reviewers. Any product that may be evaluated in this article, or claim that may be made by its manufacturer, is not guaranteed or endorsed by the publisher.
