# Supplementary material for: Manganese Dynamics in Mouse Brain After Systemic MnCl2 Administration for Activation-Induced Manganese-Enhanced MRI
Source: Front Neural Circuits. 2021 Dec 20;15:787692. doi: 10.3389/fncir.2021.787692 (PMC8722453; doi:10.3389/fncir.2021.787692)
Supplement: Supplementary file 1 [file Data_Sheet_1.pdf]

Supplementary Material

Manganese dynamics in mouse brain after systemic $MnCl_2$ administration for activation-induced manganese-enhanced MRI

Hiroki Tanihira[1], Tomonori Fujiwara[2,3], Satomi Kikuta[1,4], Noriyasu Homma[1,5], Makoto Osanai[1,6*]

[1]Department of Radiological Imaging and Informatics, Tohoku University Graduate School of Medicine, Sendai, Japan

[2]Faculty of Health and Medical Care, Saitama Medical University, Hidaka, Japan

[3]Department of Medical Physiology, Faculty of Medicine, Kyorin University, Mitaka, Japan

[4]Department of Neurophysiology, National Institute of Neuroscience, National Center of Neurology and Psychiatry, Kodaira, Japan

[5]Department of Intelligent Biomedical Systems Engineering, Graduate Scholl of Biomedical Engineering, Tohoku University, Sendai, Japan

[6]Laboratory for Physiological Functional Imaging, Department of Medical Physics and Engineering, Division of Health Sciences, Osaka University Graduate School of Medicine, Suita, Japan

*** Correspondence:**
Makoto Osanai
osanai@sahs.med.osaka-u.ac.jp

1 Supplementary Figures S1-S5

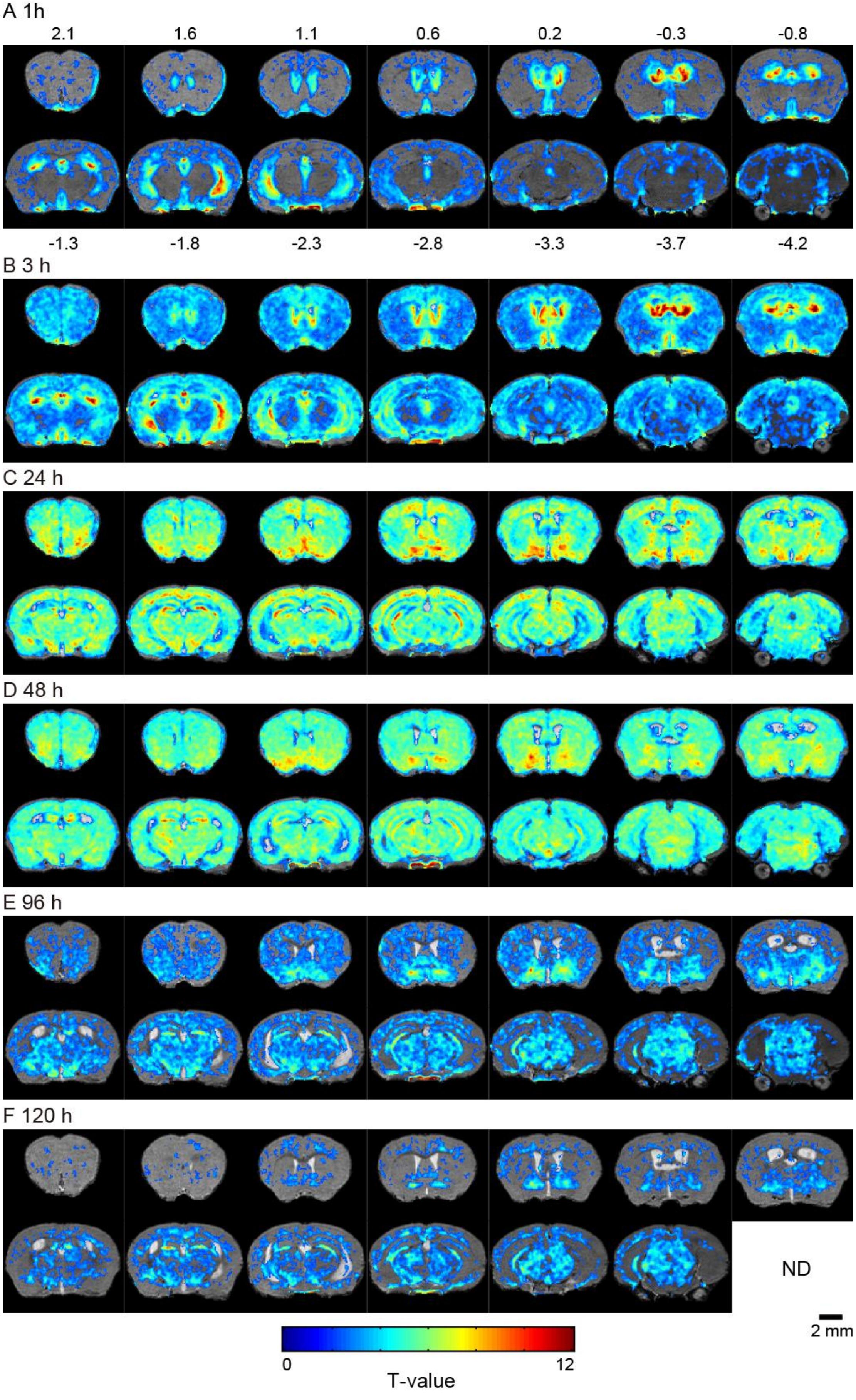
A 1h
2.1
1.6
1.1
0.6
0.2
-0.3
-0.8
-1.3
-1.8
-2.3
-2.8
-3.3
-3.7
-4.2
B 3 h
C 24 h
D 48 h
E 96 h
F 120 h
ND
2 mm
0
12
T-value

Supplementary Figure S1. Regions with significantly larger R1 after a single administration of $MnCl_2$ than before the administration are indicated by pseudo-colored regions over the brain template image. The pseudo-color map represents the distribution of *t*-values of the Student's *t*-test calculated by SPM at 1 h (A), 3 h (B), 24 h (C), 48 h (D), 96 h (E), and 120 h (F) after the administration (unpaired *t*-test, number of mice are shown in Table 1). Regions with no significant differences are not colored. Numbers shown above and below each panel in (A) indicate the anterior-posterior coordinates relative to bregma. ND: no data.

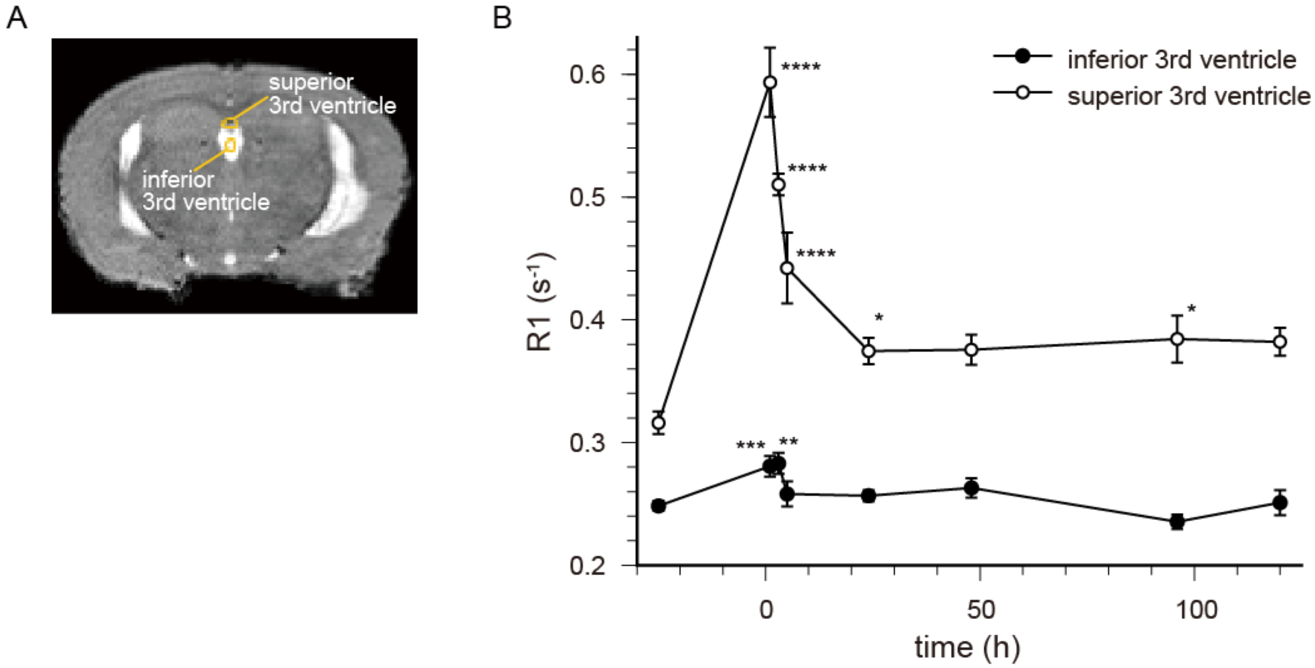

Supplementary Figure S2. [Mn^{2+}] dynamics in the superior and inferior third ventricle following a single administration of $MnCl_2$. (A) The locations of ROIs are indicated on the template MRI image. (B) Time course of the R1 values in the superior and inferior third ventricle. The horizontal axis indicates the time following $MnCl_2$ administration. Each point represents the R1 values (mean ± s.e.m) before (n=20), and 1 (n=11), 3 (n=13), 5 (n=7), 24 (n=15), 48 (n=6), 96 (n=6) and 120 (n=6) hours after the administration. *P < 0.05, **P < 0.01, ***P < 0.001, ****P < 0.0001 (Dunnet test, compared with R1 values before $MnCl_2$ administration).

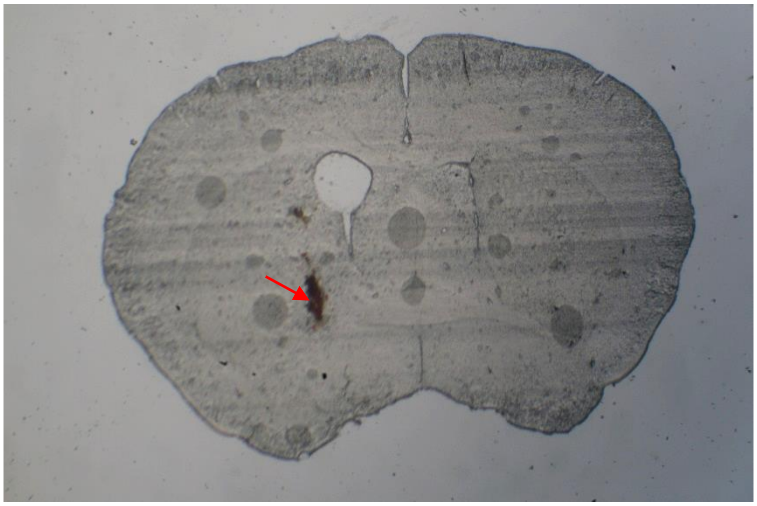

Supplementary Figure S3. The position of the dialysis probe in the *in vivo* microdialysis study (Figure 2C). One example of the probe position in the striatum. The red arrow represents the probe site in this mouse.

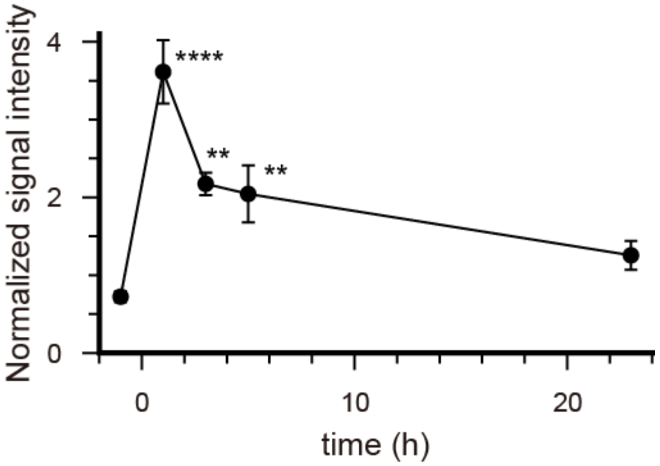

Supplementary Figure S4. Time course of the normalized signal intensity of the T1-weighted images in the gallbladder before and after $MnCl_2$ administration. **P < 0.01, ****P < 0.0001 (Dunnett test, compared with the signal intensity before $MnCl_2$ administration, n = 6)

Methods: T1-weighted images were acquired using FLASH-3D with a respiratory synchronization sequence (TR = 15 ms, TE = 3.0 ms, flip angle = 40°, FOV = 3.5 × 3.5 × 3.5 cm3, matrix size = 140 × 140 × 140). The signal intensity obtained from the gallbladder was normalized by the signal intensity of the water phantom, which was located adjacent to the mouse body.

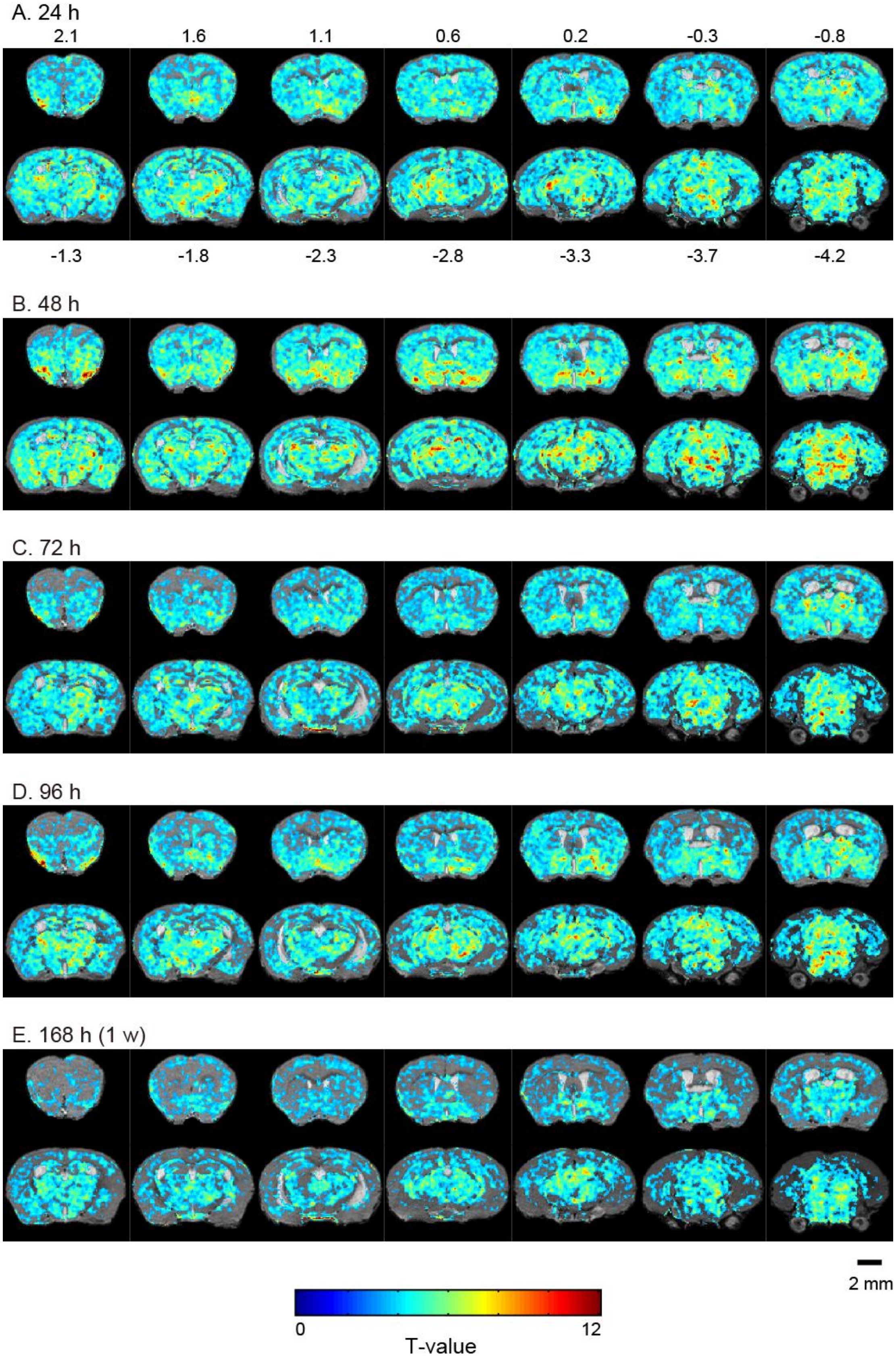
A. 24 h
2.1
1.6
1.1
0.6
0.2
-0.3
-0.8
-1.3
-1.8
-2.3
-2.8
-3.3
-3.7
-4.2
B. 48 h
C. 72 h
D. 96 h
E. 168 h (1 w)
2 mm
0
12
T-value

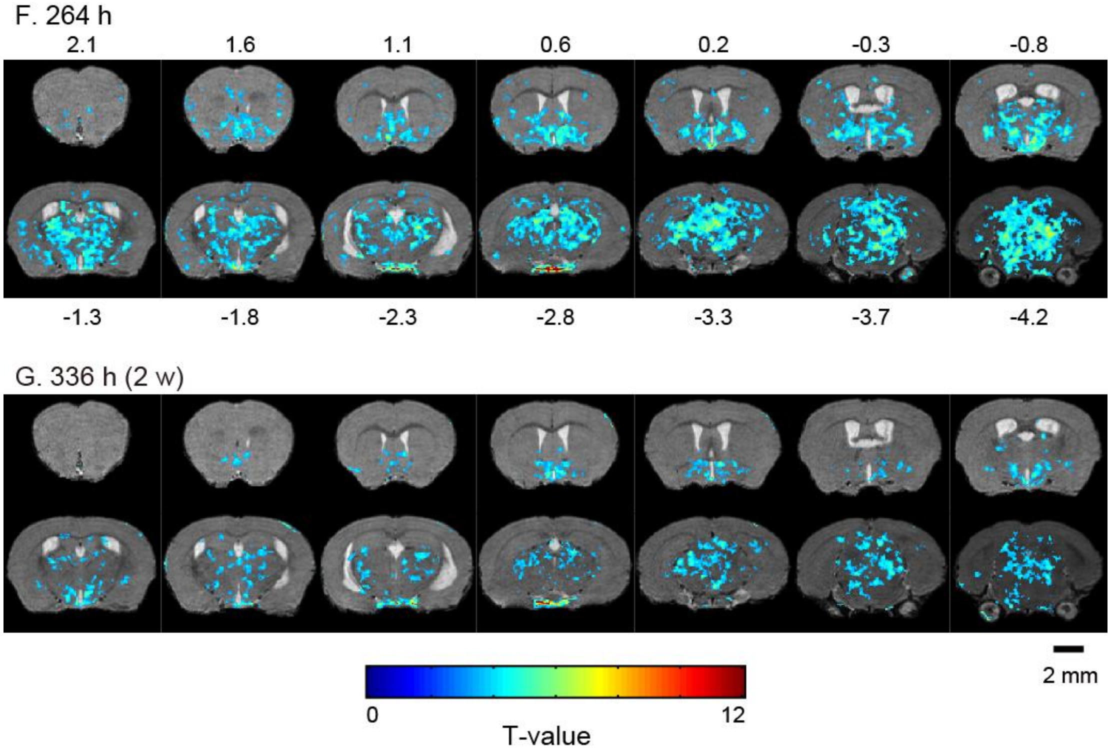

Supplementary Figure S5. Regions with significantly larger R1 after a double administration of $MnCl_2$ than before the administration are indicated by pseudo-colored regions over the brain template image. The pseudo-color map represents the distribution of *t*-values of the Student's *t*-test calculated by SPM at 24 h (A), 48 h (B), 72 h (C), 96 h (D), 168 h (1 week) (E), 264 h (F), and 336 h (2 weeks) (G) after the administration (paired *t*-test, n = 4). Regions with no significant differences are not colored. Numbers shown above and below each panel in (A) and (F) indicate the anterior-posterior coordinates relative to bregma.
